# Long-Term Use of Insomnia Medications: An Appraisal of the Current Clinical and Scientific Evidence

**DOI:** 10.3390/jcm12041629

**Published:** 2023-02-17

**Authors:** Phyllis C. Zee, Suzanne M. Bertisch, Charles M. Morin, Rafael Pelayo, Nathaniel F. Watson, John W. Winkelman, Andrew D. Krystal

**Affiliations:** 1 Department of Neurology, Center for Circadian and Sleep Medicine, Northwestern University, Chicago, IL 60611, USA; 2 Department of Medicine, Brigham and Women’s Hospital, Harvard Medical School, Boston, MA 02115, USA; 3 Department of Psychology, Cervo Brain Research Centre, Laval University, Québec City, QC G1V 0A6, Canada; 4 Department of Psychiatry and Behavioral Sciences, Stanford University Sleep Medicine Center, Redwood City, CA 94305, USA; 5 Department of Neurology, University of Washington School of Medicine, Seattle, WA 98195, USA; 6 Department of Psychiatry and Neurology, Massachusetts General Hospital, Boston, MA 02114, USA; 7 Department of Psychiatry and Neurology, UCSF Weill Institute for Neurosciences, San Francisco, CA 94158, USA

**Keywords:** insomnia, insomnia medications, safety, clinical appraisal, long-term use

## Abstract

While evidence supports the benefits of medications for the treatment of chronic insomnia, there is ongoing debate regarding their appropriate duration of use. A panel of sleep experts conducted a clinical appraisal regarding the use of insomnia medications, as it relates to the evidence supporting the focus statement, “No insomnia medication should be used on a daily basis for durations longer than 3 weeks at a time”. The panelists’ assessment was also compared to findings from a national survey of practicing physicians, psychiatrists, and sleep specialists. Survey respondents revealed a wide range of opinions regarding the appropriateness of using the US Food and Drug Administration (FDA)-approved medications for the treatment of insomnia lasting more than 3 weeks. After discussion of the literature, the panel unanimously agreed that some classes of insomnia medications, such as non-benzodiazepines hypnotics, have been shown to be effective and safe for long-term use in the appropriate clinical setting. For eszopiclone, doxepin, ramelteon and the newer class of dual orexin receptor antagonists, the FDA label does not specify that their use should be of a limited duration. Thus, an evaluation of evidence supporting the long-term safety and efficacy of newer non-benzodiazepine hypnotics is timely and should be considered in practice recommendations for the duration of pharmacologic treatment of chronic insomnia.

## 1. Introduction

Insomnia is among the most common medical conditions worldwide, affecting 15–30% of the general population [1,2,3]. The clinical diagnostic criteria defining insomnia disorder include not only symptoms of difficulty falling asleep, staying asleep and/or earlier awakening than desired for at least three times per week, but also the consequent daytime functional impairments [4]. By the time most patients with insomnia present for treatment, sleep has been disrupted for a duration of at least 3 months and thus meet the criteria for chronic insomnia disorder [5]. Women experience higher rates of insomnia compared to men [6] and the prevalence of insomnia increases in older populations [7,8,9]. Insomnia is also bi-directionally linked to mood disorders [10,11], in that insomnia is a risk factor for depression [12,13] and depression has been shown to predict the persistence of insomnia [14].

While cognitive behavioral therapy for insomnia (CBT-I) is recommended as first-line therapy, pharmacologic therapy is commonly used in the management strategy for treating insomnia [15]. Data from multiple randomized controlled trials support the efficacy of several approved pharmacological treatments including several different drug classes, such as benzodiazepine (BZD) and non-benzodiazepine (non-BZD) GABA-A modulators, dual orexin receptor antagonists (DORAs), melatonin receptor agonists and histamine antagonists [16]. Guidelines recommend a shared decision-making model when selecting pharmacologic agents for insomnia [15], with consideration of the types of patient-reported symptoms (difficulty falling asleep, staying asleep, early morning awakening), medical and psychiatric co-morbidities, concurrent pharmacotherapy, and symptom duration when weighing the risks and benefits of specific medication classes and duration of use [5]. BZDs, for example, have been associated with the potential risk of falls and cognitive impairment, as well as the potential of abuse and dependence [17,18,19,20,21]. The use of BZDs, and in particular long-term use, is not recommended in older adults due to the increased risk for these adverse events [9,22].

The recommendation by the ACP Clinical Practice Guideline on the Management of Chronic Insomnia Disorder in Adults that medication use should be limited to 4–5 weeks [15] derives from the assessment that the evidence is insufficient to adequately weigh the benefits vs. harms of the long-term use of pharmacologic therapies for chronic insomnia [23]. This view was also noted by the European Guideline for the Diagnosis and Treatment of Insomnia, which was endorsed by the World Sleep Society [24]. On the other hand, there have been studies that have examined the long-term (3 months or longer) efficacy and safety of various insomnia medications (i.e, doxepin, eszopiclone, ramelteon, lemborexant, suvorexant and daridorexant), and the American Academy of Sleep Medicine Clinical Practice Guideline for the Pharmacologic Treatment of Chronic Insomnia in Adults does not include recommendations for duration of use [5].

In view of the important ongoing dialogue regarding the appropriate duration of treatment, the availability of long-term efficacy and safety data of the newer classes of medications and the fact that insomnia is often chronic, lasting months to even years, a clinical appraisal regarding the long-term use of insomnia medications is timely. The objective of this appraisal was to evaluate the evidence for the focus statement: “No insomnia medication should be used on a daily basis for durations longer than 3 weeks at a time”. The appraisal consisted of three parts: a national survey to understand healthcare professionals’ perspectives regarding the long-term use of insomnia medications; a review of current clinical and pre-clinical literature related to the safety profile of insomnia medications; and a meeting of an insomnia expert panel to assess the evidence and implications for the practice, and to identify future needs. The literature review assessed the evidence regarding the class and mechanism of action of the insomnia medication, safety profiles and availability of short-term and long-term data.

## 2. Materials and Methods

### 2.1. National Survey

To gauge perceptions on a number of distinct but related issues regarding the management of insomnia that are the subject of current debate in the U.S. medical community, an electronic survey was distributed nationally to healthcare professionals (HCPs) (doctor-MD, doctor-PhD, nurse practitioners/physician assistants) across the United States. Responses were collected in two waves: in December 2021 and in April 2022. In wave 1: the online survey was sent to 97,000 HCPs procured from a variety of collector lists and all emails used were General Data Protection Regulation compliant. 155 responses were collected, and 47 respondents were removed as not qualified via demographic questions such as “what is your area of specialty?” and “what percentage of your time is spent managing insomnia?” The datapoint of 100 respondents was agreed to be the threshold for legitimacy for publications of this type, but given the low response rate of 0.2%, a second wave was launched in April 2022. The dataset requirements for wave 2 were the same as wave 1 and the survey was sent to an additional set of 70,319 HCPs. With a response rate of 0.6%, a further 400 eligible respondents were collected. In total, *n* = 508 eligible responses to the statement were obtained and of the 508 total the breakdown subspecialty was as follows: MDs (74%), nurse practitioners (14%), physician assistants (11%) and PhDs (1%).

Healthcare professionals involved in the management and treatment of patients with insomnia (primary care physicians, family practitioners, internal medicine specialists, psychiatrists, and sleep specialists) were asked to indicate their level of agreement with the survey statements by grading on a Likert scale from 1 to 6, with the following descriptions:

1. Strongly agree;
2. Mostly agree, but with minor reservations;
3. Slightly agree, but with major reservations;
4. Slightly disagree, due to minor reservations;
5. Mostly disagree, due to major reservations;
6. Strongly disagree.

Respondents were also given the opportunity to comment on reasons for their rating of the survey statements.

### 2.2. Expert Panel and Literature Review

The statement “No insomnia medication should be used on a daily basis for durations longer than 3 weeks at a time” was evaluated during a meeting of the expert panelists in December 2021. The statement leader presented a review of the current literature pertaining to their statement, presenting both statement-supporting and statement-refuting studies to stimulate a balanced discussion of the evidence. Further, the panelists discussed the evidence in the context of current healthcare professionals’ perceptions, as indicated by the national survey results and comments, as well as current practice.

Literature searches were performed in October 2021 for the statement “No insomnia medication should be used on a daily basis for durations longer than 3 weeks at a time” using the following electronic database: PubMed. Searches were limited to human and English and to the last 10 years. To identify relevant clinical studies, the search terms “insomnia medication daily treatment AND short term” “insomnia AND hypnotic AND continuous” and “insomnia medication prn” and “insomnia medication AND intermittent” were used, yielding 35, 46, 11 and 10 articles, respectively. Of the 102 articles retrieved, 35 articles were selected for a detailed review based on the goal of the literature search which was to identify studies that represented evidence supporting the statement and studies that provided evidence to refute the statement. Exclusions such as references presented with the criteria of US FDA-approved insomnia medication, and exclusions based on external/internal validity criteria such as study populations including a control group were applied and 20 articles were subsequently selected for presentation.

Prior to presentation of the selected studies, the panel anonymously indicated their level of agreement with the appraisal statement, using the same six-point scale used in the national survey. Following the presentation, the panel rated the quality of the literary evidence as follows:

1. Evidence obtained from meta-analysis, including at least one large, randomized control trial (RCT);
2. Evidence obtained from either meta-analysis, including at least one small RCT, or from at least one well-designed large RCT;
3. Evidence obtained from well-designed cohort or case–control studies;
4. Evidence obtained from case series, case reports, or flawed clinical trials;
5. Opinions of respected authorities based on clinical experience, descriptive studies, or reports of expert committees;
6. Insufficient evidence to form an opinion.

The panel subsequently indicated their level of agreement with the appraisal statement a second time, noting whether the data impacted their assessment, and discussed how the evidence relates to current peri-operative practice.

## 3. Survey Results and Literature Review

### 3.1. Survey Results

**Figure 1:**
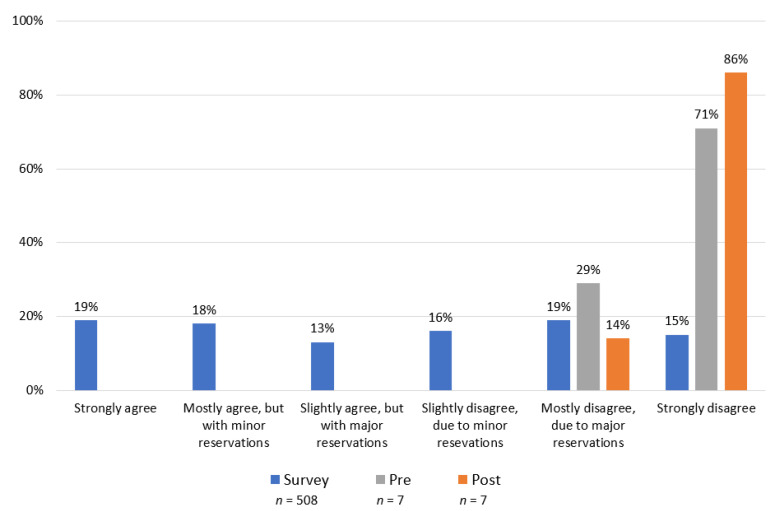
Voting results for the statement “No insomnia medication should be used on a daily basis for durations longer than 3 weeks at a time”. Survey participants *n* = 508, seven expert panelists voted before the literature search on the statement and post-presentation of the literature.

**Figure 2:**
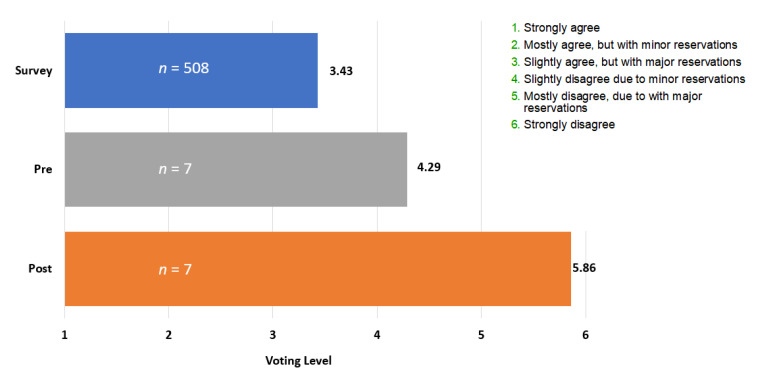
Mean Voting level for the statement “No insomnia medication should be used on a daily basis for durations longer than 3 weeks at a time”. Survey participants *n* = 508, 7 expert panelists voted before the literature search on the statement and post-presenting of the literature. 1 = strongly agree, 2 = mostly agree but with minor reservations, 3 = slightly agree but with major reservations, 4 = slightly disagree, due to minor reservations, 5 = mostly disagree, due to major reservations, 6 = strongly disagree.

For the statement “No insomnia medication should be used on a daily basis for durations longer than 3 weeks at a time” there was a wide range of responses among the survey respondents, with 50% agreeing and 50% disagreeing to some extent with the statement (Figure 1). The mean level of support among respondents was 3.20 (range 1–6; Figure 2). The most commonly cited reasons for agreeing with the statement were concerns about medication abuse and tolerance and rebound insomnia. Many also commented that prescribing insomnia medications for long-term use promotes psychological dependency. Those in disagreement with the statement mentioned that 3 weeks was too narrow of a window for some patients, that some patients appear to benefit from long-term treatment and that their patients may be reluctant to give up a medication that is helping.

In contrast with the survey respondents, all panelists expressed a prominent level of disagreement with the statement pre-presentation, and 86% strongly disagreed following the literature review (Figure 1) with their mean level of rejection of the statement strengthening after viewing the studies presented (Figure 2).

### 3.2. Safety of Insomnia Medications: Summary of the Clinical Evidence in Support of the Statement

The statement’s lead discussant presented a summary of the evidence in support of the focus statement and the review focused primarily on FDA-approved medications for insomnia. The majority of the evidence against the use of insomnia medications for longer than 3 weeks originated primarily from experience with BZDs and several non-BZDs. For BZDs, data on efficacy and safety were limited to short-term randomized controlled trials of 4 weeks or less [15,23,25,26,27,28].

Results from a meta-analysis of 24 studies (7–28 days duration and one study with triazolam for 9 weeks) on the effect of BZDs and non-BZDs in older adults with insomnia showed that while the effect on sleep improvement was very small (effect size 1.07–1.17), there was a statistically significant increase in the risk of adverse effects, such as the risk of falls and cognitive impairment [29], raising concerns about long-term use, particularly in older adults. The authors concluded that in individuals over the age of 60, the benefits of these insomnia drugs may not justify the risk.

There were a limited number of studies supporting the relationship between the long-term use of BZDs and non-BZDs, cognitive impairment and/or risk of dementia in adults, and most were retrospective and lacked a control group. In a retrospective longitudinal (28 days) study of 260,502 older adult beneficiaries of a national health registry, an increase of all-cause dementia was reported with use of BZD hypnotic medications, particularly in patients taking a short-acting BZD or multiple BZDs [30]. The lack of a longitudinal control group in this study raised the possibility that the association with dementia could be confounded by the indication (insomnia). The effects of long-term use of temazepam, zopiclone, or zolpidem on cognitive function (after medication withdrawal) was evaluated in 92 older adults [31]. Measures of attention and psychomotor performance were obtained at baseline and 1, 2 and 6 months after withdrawal of the medication. The results indicated that cognitive impairment persisted at least 6 months after withdrawal and thus the authors concluded that these drugs should be prescribed for short-term use only. However, due to the lack of a comparison group of insomnia patients who were not on hypnotic medications, the contribution of insomnia disorder to cognitive impairment remains a possibility in this study. There is evidence that, insomnia, after controlling for non-BZD hypnotics is associated with incident dementia [32] and a case–control study did not find that non-BZD use was associated with incident Alzheimer’s disease [33].

Withdrawal symptoms were examined in an open-label study of 123 patients (20–64 years of age) with insomnia disorder enrolled in a clinical trial [28] of eszopiclone (2 mg) for 24 weeks. Two weeks after discontinuation, patients completed the Benzodiazepine Hypnotics Withdrawal Symptom Scale (BHWSS), Insomnia Severity Index (ISI) and the abridged version of a self-reported Compliance and Dependence Questionnaire. There were no serious withdrawal effects, but of the 76 patients who completed the study, 8 (10.5%) experienced clinically relevant withdrawal symptoms (fatigue, weakness, nervousness, or jitteriness).

### 3.3. Safety of Insomnia Medications: Summary of the Clinical Evidence against the Statement

Following the presentation of evidence supporting the focus statement, a representative excerpt of the clinical evidence against the statement (i.e., supporting long-term use) was presented to the appraisal panel. Evidence supporting the long-term efficacy and safety of insomnia medications came from more recent studies with some of the non-benzodiazepine GABA-A modulators and newer medications with different mechanisms of action, such as ramelteon, doxepin and the dual orexin receptor antagonists.

Unlike the studies of the older class of BZDs, there is evidence of the efficacy and safety of some of the non-BZDs. In a small study [34] using objective measures of sleep (polysomnography), 33 men and women (age: 32–65) with primary insomnia without co-morbid psychiatric disorders were randomized to zolpidem at 10 mg or a placebo nightly for 12 months. Treatment capsules were color coded but not otherwise identified. 1, 2, or 3 capsules could be self-administered on any given night. Zolpidem was chosen significantly more often than the placebo. The number of zolpidem-blinded pills per night did not increase over 12 months, but the number of zolpidem pills did increase at months 4 and 12 compared to month 1. No significant withdrawal symptoms were observed on the discontinuation nights with zolpidem over 12 months. The percentage of “rebounders” did not differ between zolpidem and the placebo.

A 6-month randomized controlled clinical trial studied the effects of long-term eszopiclone [35]. The study included 830 patients with primary insomnia with a self-reported mean nightly total sleep time (TST) < or = 6.5 h/night and/or a mean nightly sleep latency (SL) > 30 min. Participants randomized to eszopiclone reported improved daytime function, including improvements in work impairment, compared to those randomized to the placebo. No significant withdrawal or rebound insomnia was reported. Adverse effects were mild, and included unpleasant taste, sleepiness, and muscle pain. Adverse effects were more frequent at the 3 mg dose of eszopiclone. A similar study on the effects of eszopiclone in elderly (65–84 years) and non-elderly adults (20–64 years) was conducted in Japan [36]. This multicenter study of 320 patients examined the effects of eszopiclone at three different doses taken for a period of 24 weeks. Results showed dose-dependent sustained improvement in self-reported sleep onset and maintenance in non-elderly and elderly patients. No rebound insomnia was observed, and adverse events were mild and dose-dependent, with the most common being unpleasant taste in both the elderly and non-elderly groups.

Efficacy and safety for longer-term use have also been studied in medications other than the GABA-A modulators. For example, a 12-week randomized, parallel, placebo-controlled study (placebo: 81; doxepin 1 mg: 77; doxepin 3 mg: 82) was conducted on the safety and efficacy of doxepin treatment in older adults (65 years and older) with insomnia [37]. Outcomes were both objective and self-reported. Significant, sustained improvement was observed in most clinical endpoints, including sleep maintenance as well as healthcare professional- and patient-reported ratings on awakening with nightly use of doxepin at 1 or 3 mg for 12 weeks. The safety profile was equivalent across the groups, and a favorable risk-to-benefit ratio was sustained over 12 weeks.

Efficacy and safety of 6-month nightly ramelteon administration in adults with chronic primary insomnia were assessed in a randomized clinical trial [38]. Over the 6 months of treatment, ramelteon reduced sleep onset (latency to persistent sleep assessed by polysomnography and subjective sleep latency) when compared to placebo with no significant next-morning residual effects, rebound insomnia or withdrawal symptoms upon discontinuation.

The newest class of medications, the dual orexin receptor antagonist (DORA) drug studies have often included data on the efficacy and safety of 3 months or longer medication use. The DORA drugs are a novel class of insomnia medications that work through a different mechanism of action compared to earlier insomnia medications. They bind to orexin receptors and inhibit the action of the wakefulness-promoting neuropeptide orexin. Suvorexant was the first DORA drug approved for treating insomnia. Long-term (1 year) suvorexant treatment was evaluated in a multi-site randomized, placebo-controlled, parallel-group clinical trial [39]. Suvorexant at 30–40 mg was determined to be efficacious for subjective measures of sleep onset and maintenance, and safe over 1 year of nightly treatment. In total 69% of the trial participants treated with suvorexant experienced adverse events, as compared 64% of those treated with the placebo. The most commonly reported adverse event was somnolence. In a 6-month placebo-controlled multicenter trial of another DORA drug, lemborexant [40], significant benefits in objective and subjective measures in sleep efficiency and wake after sleep onset were reported at 5 and 10 mg doses of lemborexant compared to a placebo. The majority of reported adverse events were mild or moderate. Daridorexant is the most recent orexin receptor antagonist approved to treat insomnia. In investigational trials, daridorexant improved sleep quality measures, as well as daytime functioning in patients with insomnia [41,42]. A randomized, double-blind, placebo-controlled trial showed that the discontinuation of daridorexant (after 3 months) among individuals with insomnia can be completed without withdrawal or rebound symptoms [43].

## 4. Discussion and Conclusions

In clinical practice, insomnia is a common chronic disorder [44,45], with long-term serious impacts on health, productivity and safety [46,47]. Although CBT-I is recommended as a first-line therapy [15], pharmacologic therapy has a vital role in the treatment of chronic insomnia. Clinicians are faced with the practical dilemma, that although insomnia is often chronic, there is a general perception that medications to treat insomnia should be limited to only short-term use of 4–5 weeks, as recommended by the ACP [15].

The purpose of this clinical appraisal exercise, therefore, was to assess the strength of the evidence supporting the views and practices of prescribing healthcare professionals (HCPs) in the field and obtain the perspectives of a panel of insomnia experts reviewing the available relevant research literature. Similar clinical appraisals [48] have served to elucidate the clinical translational gaps between the evidence-base and clinical practice to direct future dissemination activities, including educational outreach.

The focus statement for this appraisal, “No insomnia medication should be used on a daily basis for durations longer than 3 weeks at a time” contains the key caveat “No insomnia medication”. While there is no clear consensus among practitioners on how long is safe to use insomnia medications, the 3-week duration was chosen because the 1983 NIH Consensus Development Conference on Drugs and Insomnia: The Use of Medications to Promote Sleep, recommended using insomnia medications for a treatment period usually of no more than 3 weeks [49]. This was a highly influential document that has continued to influence clinical practice. Within the last 20 years, the efficacy and safety of a number of insomnia medications, such as the classes of non-BZDs, antihistamines, melatonin agonists, and DORAs, have been studied for 3 months or longer. However, current clinical guideline recommendations caution against the long-term use of insomnia medications. In practice, HCPs sometime encounter reluctance from their patient to discontinue a medication that is helping and is not reported to be associated with significant side effects.

The primary argument for the short-term use recommendation is based on the conclusion that there is insufficient evidence to weigh the benefits vs. harms of the long-term use of pharmacologic therapies for chronic insomnia [15,23,24]. In part, this derives from the lack of long-term data from efficacy and safety studies and the lack of well-controlled studies on the adverse effects or harm of long-term therapy of the older class of BZD hypnotics. Furthermore, there is concern, in older adults, that BZD hypnotics have been associated with an increased risk for adverse effects, such as impairment of balance, cognition, and psychomotor function [29,30,31]. It is important to note that the majority of these studies did not include a control group and the results may be confounded by indication; thus, causality cannot be inferred.

However, there have been long-term (6 months of longer) placebo-controlled trials of non-BZD, antihistamine, melatonin agonist, and DORA hypnotic medications that have demonstrated sustained efficacy and safety, including in older adults [34,35,36]. Recent studies with the newer MOAs (doxepin and DORAs) support long-term efficacy and safety and the use of these drugs for greater than 3 weeks nightly, yet FDA labeling does not provide any guidance for the duration of use with eszopiclone, doxepin, melatonin receptor agonists or DORAs [37,39,43].

In view of the more recent long-term studies and new classes of medications, this clinical appraisal provides an opportunity to evaluate current recommendations concerning limitations on the duration of use.

In Table 1 the data reviewed for the purposes of this clinical appraisal are summarized as medication type, study duration, and observation of adverse events. It is notable that efficacy was maintained and there were few serious adverse events in the controlled clinical trials of use for 3 months or longer.

There was a notable difference in agreement between the survey respondents and the expert panel with the focus statement, “No insomnia medication should be used on a daily basis for durations longer than 3 weeks at a time”. Exactly half of the field survey respondents agreed to some extent with the focus statement—most commonly due to concerns of abuse, tolerance and rebound of the BZ hypnotics. Half of the survey respondents reported having some reservations, but ultimately disagreed with the statement. Some respondents indicated that they had success with prescribing DORA medications long-term.

The expert panel also shared some concerns regarding adverse effects of the BZDs and non-BZDs and the limited evidence for long-term use, particularly for the BZD class of hypnotics. Despite these reservations, and after weighing the evidence, all panel members disagreed with the focus statement. Much of the basis for this disagreement was based on the fact that several clinical studies on the use of some non-BZD GABA-A modulators (zolpidem and eszopiclone) and the newer medications, such as low-dose doxepin and the DORA drugs (suvorexant, lemborexant, daridorexant), support their long-term efficacy and safety.

The conclusion of the expert panel after reviewing the clinical evidence was that the appropriate duration of pharmacologic therapy for chronic insomnia needs to be individualized based on the balance of benefits and harms—especially in older adults and patients with medical and/or psychiatric comorbidities and the use of concomitant medications. The panel also agreed that the sparsity of long-term use data prevents truly evidence-informed decision-making for the BZD drug class. Within the context of long-term use, shared decision making is therefore important, and it is recommended that patients are re-evaluated on a regular basis to assess the balance of risk-to-benefit, and that, in addition, patients are counseled on the potential utility of cognitive behavioral therapies as well as a discussion of deprescribing hypnotics.

## Figures and Tables

**Table 1:** Clinical study references presented to the expert panel for the review of relevant clinical evidence regarding the long-term use of insomnia medications. * Denotes meta-analysis. White background = evidence supporting the focus statement, light grey = evidence refuting the focus statement, pale blue = study was 24 weeks or longer.

Author (Year) [Reference]	Sample Size	Medication(s)	Mechanism of Action	Duration of Treatment
Glass et al., (2005) * [29]	24 studies, *n* = 2417	benzodiazepine (BZD), benzodiazepine receptor agonist (BZRA)	sedative hypnotic/GABA receptor agonist	7–28 days, one triazolam study lasting 9 weeks
Inoue et al., (2021) [28]	*n* = 123	BZD (eszopiclone)	sedative hypnotic/GABA receptor agonist	24 weeks
Walsh et al., (2007) [35]	*n* = 830	BZRA (eszopiclone)	sedative hypnotic/GABA receptor agonist	6 months
Mayer et al., (2009) [38]	*n* = 451	Ramelteon	melatonin receptor antagonist	6 months
Krystal et al., (2010) [37]	*n* = 240	Tricyclic antidepressant (doxepin)	serotonin–norepinephrine reuptake inhibitor	12 weeks
Roehrs et al., (2012) [34]	*n* = 33	BZRA (zolpidem)	sedative hypnotic/GABA receptor agonist	12 months
Uchimura et al., (2012) [36]	*n* = 325	BZRA (eszopiclone)	sedative hypnotic/GABA receptor agonist	24 weeks
Kärppä et al., (2020) [40]	*n* = 949	DORA (lemborexant)	orexin receptor antagonist	12 months
Michelson et al., (2014) [39]	*n* = 522	DORA (suvorexant)	orexin receptor antagonist	12 months
Kunz et al., (2023) [42]	*n* = 804	DORA (daridorexant)	orexin receptor antagonist	12 months

## Data Availability

Not applicable.

## Disclaimer

The opinions and views expressed in this publication are those of the authors and do not necessarily reflect the opinions and recommendations of the publisher or supporters. This publication is intended for scientific exchange purposes and results from an evidence-based collaborative process in which all authors may not agree on all points. Pharmaceutical products and/or use of products that have not been approved by the US Food and Drug Administration or other regulatory authorities may be discussed. Dosages, indication, and methods of use for compounds that are referred to by the authors may reflect their clinical experience or may be derived from the professional literature or other sources. For approved product information, consult the manufacturer’s prescribing information or the applicable regulatory authority.
